# An Evaluation of Norspermidine on Anti-fungal Effect on Mature *Candida albicans* Biofilms and Angiogenesis Potential of Dental Pulp Stem Cells

**DOI:** 10.3389/fbioe.2020.00948

**Published:** 2020-08-12

**Authors:** Yan He, Yanfan Cao, Yangfan Xiang, Fengting Hu, Fengyu Tang, Yanni Zhang, Abdullkhaleg Ali Albashari, Zhenjie Xing, Lihua Luo, Yan Sun, Qiang Huang, Qingsong Ye, Keke Zhang

**Affiliations:** ^1^Laboratory for Regenerative Medicine, Tianyou Hospital, Wuhan University of Science and Technology, Wuhan, China; ^2^School & Hospital of Stomatology, Wenzhou Medical University, Wenzhou, China; ^3^Department of Pediatric Dentistry, School and Hospital of Stomatology, Wenzhou Medical University, Wenzhou, China; ^4^School of Stomatology and Medicine, Foshan University, Foshan, China; ^5^Center of Regenerative Medicine, Renmin Hospital of Wuhan University, Wuhan, China

**Keywords:** norspermidine, *Candida albicans*, regenerative dentistry, anti-fungal effect, dental pulp stem cells, differentiation

## Abstract

Norspermidine (Nspd) is a kind of polyamine molecule, which is common in eukaryotes and prokaryotes. It has been reported as a potential anti-biofilms agent of bacteria, but its anti-fungal effect remains unclear. *Candida albicans* (*C. albicans*) is a common opportunistic pathogen in oral cavity of human beings. *C. albicans* biofilm is often seen in dental caries. In this work, we aimed to study the effect of Nspd on mature *Candida albicans* biofilms and to investigate how Nspd would influence human dental pulp stem cells (DPSCs). Our biofilm assays indicated that 111.7 and 55.9 mM Nspd dispersed 48 h mature fungal biofilms and showed significant fungicidal effect. 27.9 and 14.0 mM Nspd showed moderate fungicidal effect. Live/dead staining echoed the fungicidal effect. 111.7–14.0 mM Nspd showed a dose- inhibitory effect on mature fungal biofilm, where 14.0 mM Nspd reduced the metabolic activity by half compared with blank control. Moreover, we demonstrated that 111.7–27.9 mM Nspd restrained the production of hyphae form of *C. albicans* via SEM. Low dose Nspd (27.9 and 14.0 mM) could significantly reduce virulence related gene expression *in C. albicans* biofilms. MTT assay displayed a dose effect relation between 2.5–0.08 mM Nspd and DPSCs viability, where 0.63 mM Nspd reduced the viable level of DPSCs to 75% compared with blank control. Live/dead staining of DPSCs did not show distinctive difference between 0.63 mM Nspd and blank control. Vascular differentiation assay showed capillary-like structure of inducted DPSCs culture with and without 0.63 mM Nspd suggesting that it did not significantly affect angiogenic differentiation of DPSCs. Nspd can penetrate remaining dentin at low level, which is confirmed by an *in vitro* caries model. In conclusion, our study indicated high dosage Nspd (111.7 and 55.9 mM) could effectively disrupt and kill mature fungal biofilms. Low dosage (27.9 and 14.0 mM) showed mild anti-fungal effect on mature *C. albicans* biofilms. Human DPSCs were tolerate to 0.08–0.63 mM Nspd, where viability was over 75%. 0.63 mM Nspd did not affect the proliferation and angiogenetic differentiation of DPSCs.

## Introduction

*Candida albicans* (*C. albicans*) is widespread in normal human microbiota and it is one of the most important fungi colonies asymptomatically residing in oral cavity (Mayer et al., 2013). It is also an opportunistic pathogen that can cause infection in immunocompromised individuals under certain circumstances and in healthy individuals with implanted medical and dental devices (Bachtiar et al., 2016; Lin et al., 2019). It can cause skin diseases, vaginal mucosal infections, meningitis, and systemic diseases when immunological functioning is disturbed (Nobile and Johnson, 2015). Also it is the main pathogen causing denture stomatitis and compromising the oral function. *C. albicans* adhered to dental surfaces and forms biofilms, it was closely related to cariogenic microbe (Liu et al., 2017). Investigations indicated that *C. albicans* has been frequently found in early childhood caries (Yang et al., 2012). There are other possible reasons causing caries in childhood, such as fixed orthodontic treatments (Hu et al., 2013). Morphological transition of *C. albicans* in dimorphic, hydrolytic enzymes secretion, invasins, thigmotropism and phenotypic switching, adhesion and biofilms formation are regard as virulence to make a contribution to its pathogenesis (Soll and Daniels, 2016; Solis et al., 2018). It is reported that the biofilm mode of *C. albicans* is responsible to most fungal infections in clinical settings, which anchors on surfaces of medical implants and spreads to remote tissue and organs via blood circulation. Biofilm formation starts with the adherence of yeast-form cells on surface, followed by proliferation of yeast-form cells (Ishchuk et al., 2019). Usually mature biofilm forms a robust and complicated structure with yeast, pseudohyphae and hyphae wrapped in extracellular matrix (Finkel and Mitchell, 2011; Metwalli et al., 2013).

Common drugs to treat the fungal infection clinically are fluconazole, amphotericin B and Caspofungin acetate, which can inhibit the formation of *C. albicans* biofilm at varying degrees (Uppuluri et al., 2011). Nevertheless, conventional infection control of *C. albicans* biofilm is ineffective in that a mature fungal biofilm is more resistant to these drugs and arrantly hard to be eradicated, which requires higher dosage and results in severe side effects and excessive medical expenditure (Van et al., 2018). Due to the structural characteristics, extracellular matrix of *C. albicans* biofilm could isolate anti-fungal drugs acting as barriers. The extracellular DNA and polysaccharides in the matrix were found to contribute to anti-fungal resistance (Davey and O’toole, 2000; Bjarnsholt et al., 2013). *C. albicans* cells in biofilm model are more resistant to anti-biotics compared with the planktonic fungi, up to more than 1,000 times higher (Tobudic et al., 2012). Moreover, the emergence of drug-resistant strains made it more difficult to treat the infections. It is very urgent to develop new anti-fungal or anti-biofilms agents in the post-anti-biotic era (Srivastava et al., 2018).

Polyamine was initially found to affect the activity of cyclin-dependent kinases during the DNA replication to regulate cell proliferation and it also had aliphatic groups that influence cell growth, proliferation, division and differentiation, as well as membrane stability (Oredsson, 2003; Igarashi and Kashiwagi, 2010). Norspermidine (Nspd), one kind of polyamines, has drawn an increasing attention for its potential against bacterial biofilms recently. The effect of Nspd on bacterial biofilms was species dependent and dose dependent. It showed inhibitory effects biofilms of many pathogens including *Pseudomonas aeruginosa* (*P. aeruginosa*), *Acinetobacter baumannii* (*A. baumannii*), *Staphylococcus epidermidis* (*S. epidermidis*), *Streptococcus mutans* (*S. mutans*), *Salmonella* and *Klebsiella pneumonia* (*K. pneumoniae*) (Figueiredo and Ferreira, 2014; Konai et al., 2014; Nesse et al., 2015; Qu et al., 2016; Wu et al., 2016; Ou and Ling, 2017; Sun et al., 2019). Nspd was displayed to show inhibition on planktonic form of *C. albicans* in polyamine free medium (Hamana et al., 1989). Being able to form biofilms is one of the most important virulence factors of *C. albicans.* Factors such as complicated biofilm architecture, extracellular matrix, enhanced expression of drug efflux pumps and metabolic plasticity made contributions to enhance the drug resistance of a mature fungal biofilm (Lynch and Robertson, 2008; Wu et al., 2015). Up to date, there was no knowledge about the effect of Nspd on fungal biofilms.

Deep caries irritates dental pulp and thus influence the dental pulp vitality. Dental pulp stem cells have become a promising source of stem cells. Dental pulp preservation has been increasingly important. Dental pulp stem cells (DPSCs) derived from the neural crest carry the characteristics of mesenchymal stem-ness. DPSCs and other odontogenic stem cells can proliferate and differentiate into multiple tissue cells (Chen et al., 2013; Luo et al., 2018). Therefore, DPSCs have been widely used in the field of regenerative medicine research, preclinical research, which includes oral diseases (Morsczeck et al., 2005); Also advanced technology, such as CBCT can assist the evaluation of the regeneration outcomes (Sun et al., 2014). Studies have showed various good regenerative potency of DPSC when co-cultured with drugs and scaffolds. In our study, Nspd was targeted to treat fungal infection caused by *C. albicans*. when treating dental caries with Nspd, it is important to assess the effect of Nspd on the proliferation and differentiation of DPSCs.

## Materials and Methods

### Organism, Growth Condition

*Candida albicans* SC5314 (*C. albicans*) was obtained from the Institute of Stomatology (Wenzhou Medical University, China). *C. albicans* was cultured overnight routinely in Sabouraud Dextrose Broth (SDB; Solarbio Science & Technology Co., Ltd., China) for proliferation in aerobic circumstances. Morpholinepropanesulfonic acid (MOPS; Solarbio Science & Technology Co., Ltd., China) modified RPMI-1640 media (Thermo Fisher Scientific, United States) was used to support the minimal inhibitory concentration (MIC) assay and biofilm associated experiments. MIC was defined as the concentration at which *C. albicans* growth was visibly inhibited. The fungi and biofilms were statically cultured at 37°C aerobically (Oredsson, 2003).

For biofilm associated experiments, RPMI-1640 medium contained no Nspd was set as blank control, 10 × MIC of fluconazole against *C. albicans* SC5314 was used as positive control and 0.08% ethanol was set as vehicle control, as ethanol was used as solvent in preparing fluconazole (Seleem et al., 2016). The working cell densities of fungi were 5 × 10^3^ and 5 × 10^5^CFU/ml for MIC of fluconazole against *C. albicans* and 48 h *C. albicans* biofilm formation, respectively. 96 well plates were used to culture *C. albicans* biofilms in most studies. If biofilms were formed in other system, specific description would be made to clarify. Nspd (Sigma-Aldrich Corporation, United States) dissolved in MOPS modified RPMI-1640 media was used to culture 48h old *C. albicans* biofilm for another 48h to investigate the effect of Nspd on mature *C. albicans* biofilms.

### Biomass Assay

To investigate the effect of Nspd on *C. albicans* biofilm biomass, crystal violet assay was conducted as previously described (Peeters et al., 2008; Huo et al., 2011). Nspd (111.7, 55.9, 27.9, and 14.0 mM) conditioned growth media were added to 48h pre-formed *C. albicans* biofilms separately in 96-well plates and cultured for another 48h. Then biofilms were stained with 0.1% (w/v) crystal violet for qualitative analysis by capturing the biofilms images using a stereoscope (Nikon Corporation, Tokyo, Japan). To quantitatively assess the biomass, biofilms retained crystal violet was dislodged with 150 μl of 33.3% (v/v) acetic acid (Zhongxing Chemical reagent Co., Ltd., Zhejiang, China) for 10 min. 100 μl of dissolved solution were added to a new plate. Then absorbance was measured at 595 nm by a microplate reader (Thermo Fisher Scientific, United States).

### Fungicidal Assessment

Colony forming unit (CFU) count was used to evaluate the fungicidal effect of Nspd on *C. albicans* biofilms. After 48 h Nspd treatment on the biofilms, phosphate buffer saline (PBS) was used to remove loosely attached cells. Fungi were thoroughly scraped off from the bottom of culture plates and re-suspended in 200 μl of sterile PBS by vortex. For CFU count, 10-fold gradient dilution was prepared and 100 μl *C. albicans* diluted suspension was inoculated on Sabouraud’s Agar plates (SDA; Solarbio Science & Technology Co., Ltd., China) and cultured 24h before colony counting (Seneviratne et al., 2009). Log_10_ CFU/ml was calculated for the fungicidal comparison.

### Fungal Metabolic Assessment – XTT Assay

2, 3-bis (2-methoxy-4-nitro-5-sulfo-phenyl)-2H-tetrazolium-5-carboxanilide (XTT; Invitrogen, Carlsbad, CA, United States) assay was used to detect the metabolic activity of *C. albicans* biofilms influenced by Nspd (Ramage et al., 2001). After washed twice with PBS, the biofilm was incubated with 150 μl XTT solution (0.5 mg/ml) at 37°C in darkness for 1 h. 100 μl of incubated solution were extracted for absorbance measurement at 490 nm by the microplate reader.

### Live/Dead Staining of *C. albicans*

To detect the influence of Nspd on the vitality of *C. albicans* inside biofilms, live/dead staining was conducted using a LIVE/DEAD^®^ BacLight^TM^ Bacterial Viability Kits (Invitrogen Carlsbad, CA, United States) following the manufacturer’s instruction as previous (Samaranayake et al., 2009). Briefly, biofilms were stained with SYTO 9 and propidium iodide (PI) at 37°C for 30 min in the darkness, a confocal laser scanning microscope (Nikon Corporation, Tokyo, Japan) was used to acquire biofilm images at 3 random sites on each sample using a 60 × oil immersion lens. The excitation/emission wavelength was set at 480/500 nm for SYTO 9 and 490/635 nm for PI.

### Scanning Electron Microscope Imaging

To observe the morphological features of the biofilms by SEM, 10 mm × 10 mm × 3 mm acrylic resin chips (Jianchi Dental Equipment, Changzhi, China) were purchased and sterilized by ethylene oxide (Silva S. et al., 2013; Bachtiar et al., 2016). The acrylic resin chips were placed into 24-well plate to form 48h old *C. albicans* biofilms. Various dosages of Nspd were co-cultured with 48h old biofilms for another 48h. Biofilms were washed twice with PBS, fixed with 2.5% (w/v) glutaraldehyde (J&K Scientific, Co., Ltd., China) and dehydrated by gradient ethanol (Sangetha et al., 2009). The samples were sputter-coated with gold-palladium. Biofilm structure and the fungal morphology were imaged by SEM (Hitachi, Tokyo, Japan). The captured images were presented at 2000 × magnification.

### Genes Expression

To explore the mechanism how Nspd influence the formation of biofilm, the expression level of *C. albicans* hyphal wall protein1 (*hwp1*), agglutinin-like sequence protein 3 (*als3)* and cell surface hydrophobicity (*csh1*) were studied. Hyphal proliferation and substratum adhesion related protein, Hwp1, plays a decisive role in *C. albicans* adhesion, virulence expression and pathogenesis (Feldman et al., 2016). The expression of *als3*, a hyphae-specific cell surface protein, played a decisive role in the adhesion of *C. albicans* biofilms formation (Feldman et al., 2016). The *csh1* is the first gene that has been proved to be important in the production of cell surface hydrophobicity mucin and it acts vitally in biofilms formation (Feldman et al., 2016).

#### RNA Isolation

14.0 and 27.9 mM Nspd were selected to challenge the 48 h old biofilms for 48 h. Then the biofilms were collected for RNA extraction using Trizol (Theiss et al., 2006). Concentration and purity of the extracted RNA were verified by Nanodrop 2000, and the integrity of RNA was confirmed by agarose gel electrophoresis.

#### Reverse Transcription

Reverse transcription was conducted to obtain cDNA by a PrimeScript^TM^ RT reagent Kit with gDNA Eraser (Perfect Real Time) (Takara Bio Inc., Otsu, Japan) following the manufacturer’s instructions.

#### Real-Time Quantitative PCR

TB Green^®^ Premix Ex Taq^TM^ II kit (Takara Bio Inc., Otsu, Japan) was used for qRT- PCR analysis (Li et al., 2018). The total reaction volume was 20.0 μl (10.0 μl 2 × SYBR Premix Ex Taq II, 0.8 μl forward primer, 0.8 μl reverse primer 0.4 μl reference dye II, 2.0 μl of cDNA and 6.0 μl sterilized distilled water). The reaction procedure was set as follows: pre-denaturation at 95°C for 30s, followed by 40 cycles of denaturation at 95°C for 5s, annealing at 55°C for 30s, extension at 72°C for 30s in a StepOnes plus (Life Technologies, United States). The results were analyzed by 2^–ΔΔ*Ct*^ method. *18S rRNA* was used as reference gene. Primers were: *18S rRNA* (F: 5′-CACGACGGAGTTTCACAAGA-3′; R: 5′-CGATG GAAGTTTGAGGCAAT-3′), *hwp1* (F: 5′-GCTCCTGCTCCTGA AATGAC-3′; R: 5′-CTGGAGCAATTGGTGAGGTT-3′), *als3* (F: 5′-CAACTTGGGTTATTGAAACAAAAACA-3′; R: 5′-AG AAACAGAAACCCAAGAACAACC-3′), *csh1* (F: 5′-CTGTC GGTACTATGAGATTG-3′; R: 5′-GATGAATAAACCCAA CAACT-3′) (Feldman et al., 2016).

### Cell Culture, Identification and Multilineage Differentiation

DPSCs were extracted, cultured and identified in accordance with our previous study (Luo et al., 2018). The use of DPSCs and protocols used in this study was independently reviewed and approved by the Ethics Committee of the School and Hospital of Stomatology, Wenzhou Medical University (No. WYKQ2018008). Cells from passage 3–5 were used in this study. 2 × 10^3^ cells/well was seeded in the 96-well plates and 100 μl per well medium was added. If other seeding density or culture system were applied, separate description would be made. Complete α-modified Eagle’s medium (α-MEM, Thermo Fisher Scientific, United States) was prepared with addition of 10% fetal bovine serum (FBS, Thermo Fisher Scientific, United States) and 1% antibiotics [100 IU/mL penicillin (Thermo Fisher Scientific, United States) and streptomycin (Thermo Fisher Scientific, United States)]. This complete α-MEM was also used as control. DPSCs were cultured at 37°C in 5% CO_2_ 70% humidified incubator. DPSCs were prepared in triplicate to minimize result variation.

Flow cytometry analysis was applied to confirm the immunophenotyping of DPSCs used in this study. When 80–90% confluence reached, stem cells were incubated with the following monoclonal antibodies: CD73, CD105, CD34, and CD45 (BioLegend, United States) for 30 min at 4°C in the darkness. Stained cells were washed three times and re-suspended in PBS with 1% BSA. Then the stem cells were analyzed with CytoFLEX flow cytometer (Beckman Coulter, California, United States).

Adipogenic differentiation: DPSCs were plated into 6-well plates with a density of 1.5 × 10^5^ cells/well. When 100% confluence or post-confluence was achieved, OriCell TM mesenchymal stem cells adipogenic differentiation medium (Cyagen, United States) was used to induce adipogenic differentiation of DPSCs according to manufacturer’s instructions. After 21 days of differentiation, cells were fixed with 4% PFA for 20 min and stained with oil red O for half an hour to identify the lipid droplets in adipose cells. Staining result was observed and analyzed by light microscope (TS100, Nikon, Japan).

Osteogenic differentiation: DPSCs were plated into 6-well plates with a density of 1.5 × 10^5^ cells/well. Until 60—70% confluence, the medium was replaced with OriCell TM mesenchymal stem cell osteogenic differentiation medium (Cyagen, United States) and cultured for 21 days. Culture medium was renewed twice a week. After 3 weeks of induction, the cells were washed with PBS and fixed with 4% PFA for 20 min, then stained with alizarin red S (Cyagen, United States) at room temperature in the dark for 3–5 min to identify calcified tissue. Staining result was observed and analyzed by light microscope (TS100, Nikon, Japan).

Chondrogenic differentiation: 2.5 × 10^5^ cells were collected by centrifugation at 1,000 rpm for 5 min. Then the cell pellets were cultured in chondrogenic medium in a humidified atmosphere with 37°C, 5% CO_2_ for 28 days. During differentiation, culture medium was changed every 3 days. At the end of chondrogenic pellets’ formation, tissue pellet was fixed with 4% PFA for 20 min and stained with Alcian blue to identify differentiated chondrocytes. Staining result was observed and analyzed by light microscope (TS100, Nikon, Japan).

### DPSCs Metabolic Activity Assessment – MTT Assay

MTT assay was to define the cytotoxicity and determine the optimal concentration of Nspd for further experiments. The old media of 24h culture were replaced with complete α-MEM containing FBS, 1% antibiotics, streptomycin and added various concentrations of Nspd (0.0, 0.08, 0.16, 0.31, 0.63, 1.25, 2.5, 5.0 mM). Cell culture with the presence of Nspd was continued for 24h. Then the medium was removed and washed twice with PBS. 100 μl per well 0.5 mg/mL MTT solution was added and incubated at 37°C for 2h. The MTT solution was discarding and rinsed by PBS. 150 μl per well DMSO was used to dissolve the formation of crystals and transferred to a new plate. Optical density (OD) values of solution were measured photometrically at 570 nm by an absorbance microplate reader (Vikas et al., 2019).

### Live/Dead Staining of DPSCs

To compare the vitality of DPSCs with and without the presence of low dosage of Nspd, live/dead staining was used to treat DPSCs that were cultured with serially diluted Nspd for 24h. Old media were removed and washed twice with PBS. A blend of 3 μl PI (1 mg/ml), 2 μl of Calcein AM (1 mg/ml) and 1 ml PBS was prepared and aliquoted. 100 μl per well of the mixture was added and incubated for 30 min in the dark at room temperature. PBS was used to replace the stain. Fluorescence microscope (Axiovert A1, Carl Zeiss, Germany) was used to visualize and capture images. Live cells exhibited green, and the dead appeared red (Yao and Flynn, 2018).

### Capillary-Like Network Formation

To investigate the effect of Nspd on angiogenic differentiation of DPSCs, GelMA hydrogels was used as a scaffold structure (Chen et al., 2012). Complete endothelial cell growth medium-2^TM^ (EGM-2^TM^, containing 2% FBS, 0.4% hFGF-B, 0.1% VEGF, 0.1% hEGF, 0.1% R3-IGF-1, 0.1% Heparin, 0.1% ascorbic acid, 0.1% gentamicin/amphotericin-B, and 0.04% hydrocortisone) (Lonza Bioscience, Switzerland) with various concentrations of Nspd were added to the confluent DPSCs and GelMA in 96-well plates. After 7 day differentiation, cells were washed with PBS and fixed with 4% PFA for 15 min at room temperature. Cells were permeabilized with 0.1% Triton X-100 for 20 min and washed three times in PBS. Then the cells were incubated with Phalloidin-TRITC (Solarbio Science & Technology Co., Ltd., China) (1:200 with 1% BSA) for 1h in the dark at 37°C, followed by incubation with DAPI for 5 min. Phalloidin-TRITC was used to stain F-actin, one of the cytoskeletons, which is important to cell-to-cell and cell-to-matrix adhesion. DAPI was used to stain nuclei. The samples were analyzed through fluorescence microscopy (Nam et al., 2017).

### Dental Caries Model

To study whether Nspd would permeate into pulp chamber and affect DPSCs inside the chamber, we introduced a dental caries model with teeth decay on the surface *in vitro* (Figure 7A, arrow on the right). The use of teeth and protocols described in this study was approved by the Ethics Committee of the School and Hospital of Stomatology, Wenzhou Medical University (No. WYKQ2020002). The pulp tissue was removed from extracted teeth via lateral access (Figure 7A, arrow on the left). Saline (100 μl per teeth in dental pulp tune) was added inside the pulp chamber. All access to external environment including the apical foramina was sealed. Then teeth (1 tooth/well) were placed in 24-well plate containing 116.7 mM of Nspd. The teeth were incubated at 37°C for 48 h.

#### Quantification of Nspd

After 48 h, liquid from sealed dental pulp chamber was extracted. To quantify the Nspd by gas chromatography (GC; Agilent Technologies, United States), the sample was diluted 250 times with methanol, recorded as dental samples. A standard Nspd solution was prepared with methanol to make Nspd at 330.68 ng/ml, served as control and recorded as substance group. Different dental samples and substance were detected by gas chromatography analysis (GC; Agilent Technologies, United States). Linear velocity of helium carrier gas was 40.0 cm/sec; Oxygen flow rate was 66.7 ml/min; Hydrogen flow rate was 16.7 ml/min; Nitrogen as auxiliary gas; split ratio was 39:1; Injector temperature was 280°C; Initial column temperature was 120°C and at a hating rate of 25°C/min to 260°C; The temperature of gasify room and detector were 300°C (Retention time: 10 min).

### Statistical Analysis

One-way Analysis of Variance (ANOVA) was used to analyze the data, followed by the Tukey’s multiple comparison tests. Statistical significance was set as *p* < 0.05.

## Results and Discussion

### MIC of Fluconazole for *C. albicans*

The MIC of fluconazole against *C. albicans* was 8 μM. 80 μM fluconazole was used as positive control.

### Nspd Reduced the Biomass of Mature *C. albicans* Biofilms

**FIGURE 1:**
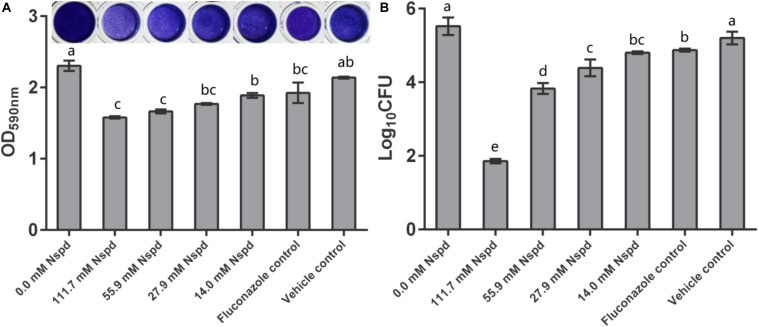
*C. albicans* biofilm biomass determined by crystal violet assay and CFU count. **(A)** Quantitative analysis of biofilm biomass by crystal violet assay with inserted panel of representative *C. albicans* biofilm stained with crystal violet. 111.7 and 55.9 mM Nspd showed obvious removal effect on 48 h old mature fungal biofilms. Other Nspd, positive and vehicle controls showed moderate biofilm removal effect. **(B)** CFU count of *C. albicans* in biofilms after 48 h exposure to interventions. CFU counting indicated survival proportion of *C. albicans* in biofilm. 111.7 and 55.9 mM Nspd showed significant fungicidal effect on mature biofilms. Other Nspd and positive control groups showed mild fungicidal effect. Vehicle control, containing 0.08% ethanol, showed no fungicidal effect on mature biofilms. Data were presented in mean ± standard deviation and values with dissimilar letters were significantly different from each other; *p* < *0.05*.

Crystal violet staining results showed that Nspd inhibited the biomass of mature *C. albicans* biofilms in a dose dependent manner. Nspd showed more inhibitory effect on pre-formed biofilms than fluconazole did, which merely reduced 16.44% when compared with blank control. 111.7 and 55.9 mM Nspd significantly reduced 31.45% and 27.80% biomass of the biofilm (*p* < 0.05) (Figure 1A). Vehicle group hardly affected the biomass (*p* > 0.05) indicating the reduction of biomass was contributed by Nspd.

According to previous studies, Nspd was suggested to disassemble mature biofilms through targeting extracellular polysaccharide in a large, old-aged microbial aggregates model (Si et al., 2014). Cardile et al. (2017) claimed 20 mM Nspd was effective in dispersing and inhibiting 24 h MRSA bacterial biofilm. However, the actual mechanism of the anti-biofilms effect of Nspd on *C. albicans* remained unknown. As cell structure of *C. albicans* is quite different from that of bacteria, the molecular mechanism of Nspd affecting the *C. albicans* biofilms might differ from that of bacteria.

### Fungicidal Assessment

Colony forming unit results showed fungicidal effect of Nspd on pre-formed *C. albicans* biofilms. 14.0, 27.9, 55.9 and 111.7 mM Nspd significantly reduced 12.98%, 20.50%, 30.64%, and 66.37% Log_10_ CFU, respectively, when compared with blank control (Figure 1B). The fungal count was significantly decreased in Nspd treated at the concentration of 111.7 mM when compared to lower concentrations of Nspd, fluconazole and vehicle control groups (*p* < 0.05). Fluconazole only reduced 11.72% Log_10_ CFU when compared with the blank control group and showed no significant difference to 14.0 mM Nspd group (*p* > 0.05). Vehicle control showed no significant fungicidal effect (*p* > 0.05). The fungicidal ability of Nspd was significantly enhanced at 111.7 mM concentration when compared to other experiment and control groups. Antimicrobial effect of Nspd varied from bacterial strains, Cardile et al. studied the antimicrobial effect of 20 mM Nspd on planktonic bacterial growth in liquid media for 24 h. They reported that 20 mM Nspd could effectively kill *S. aureus*, could suppress the growth of *A. baumanii* and *K. pneumoniae*, and could not affect *P. aeruginosa* (Cardile et al., 2017).

### Nspd Reduced the Viability and Metabolic Activity of *C. albicans* Biofilms

**FIGURE 2:**
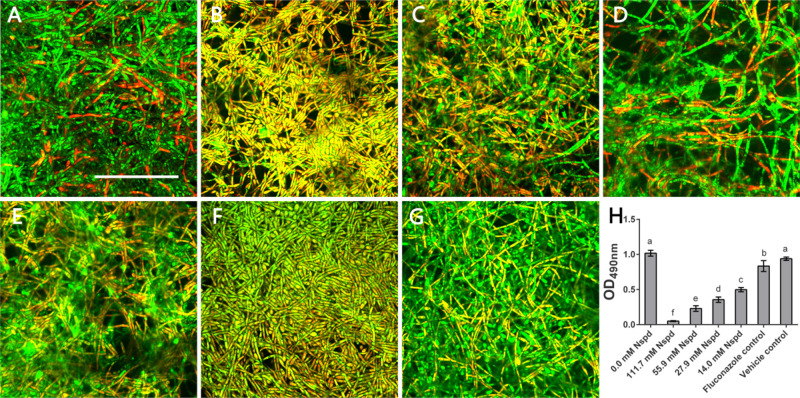
Live/Dead staining and metabolic activity of *C. albicans* biofilms. Representative *C. albicans* biofilm images revealed by live/dead staining: **(A)** Blank control group (0 mM Nspd) showed few dead cells in a mature biofilm. **(B–E)** The number of dead cells (red) decreased as Nspd was diluted from 111.7 to 14.0 mM. **(F)** 80 μM fluconazole group and **(G)** vehicle group (0.08% ethanol), both displayed a few dead cells inside the biofilm. Live cells appeared as green and dead cells appeared as red; yellow/orange colors were the result of an overlap of fluorescent dyes; bar = 50 μm. **(H)** Metabolic activity of *C. albicans* biofilms was determined by XTT assay. A clear dosage effect relation was displayed in the metabolic level and dilution of Nspd. Nspd could significantly suppressed the metabolic activity of fungal cells in mature biofilm, much more effective than positive and vehicle controls. 14.0 mM Nspd could inhibit the metabolic activity level of mature fungal biofilm to half of the blank control’s (0.0 mM). Data were presented in mean ± standard deviation and values with dissimilar letters were significantly different from each other; *p* < 0.05.

According to live/dead staining results, Nspd reduced the viability of *C. albicans* biofilms. There was more dead *C. albicans* (stained red) in Nspd containing groups when compared to blank control group (Figure 2). Fluconazole and vehicle control group could not inhibit *C. albicans* biofilms which had less dead fungus (Figures 2A,G). The XTT results showed that Nspd showed significant inhibitory effect on the metabolic activity of *C. albicans* biofilms in a dose dependent manner when compared with bank control (*p* < 0.05) (Figure 2H). Fluconazole group affected metabolic activity of *C. albicans* in biofilm to a much less degree than Nspd containing groups did (*p* < 0.05), which only reduced by 18.1% when compared with blank control. There was no significant difference between blank control and vehicle control. Hamana et al. (1989) reported that planktonic *C. albicans* did not grow with presence of 100 μM Nspd for 48 h. In our study, to effectively inhibit the vitality of *C. albicans* in mature biofilm, only 111.7 mM Nspd was successful after 48 h (Figure 1B). This suggested that biofilm mode of fungi did increase the resistance of antibiotics greatly.

The viability and metabolic activity are important for virulence effect of biofilm. Biofilm featuring the multicellular and complex structures may provide prevention strategies for fungus (Ramage et al., 2001). Yeast phase of *C. albicans* was a probable way of self-protection. We noted that compared with fluconazole, high dosage Nspd effectively inhibited the viability and metabolic activity of mature fungal biofilm.

### Npsd Induced More Yeast Phase of *C. albicans* in Biofilm

**FIGURE 3:**
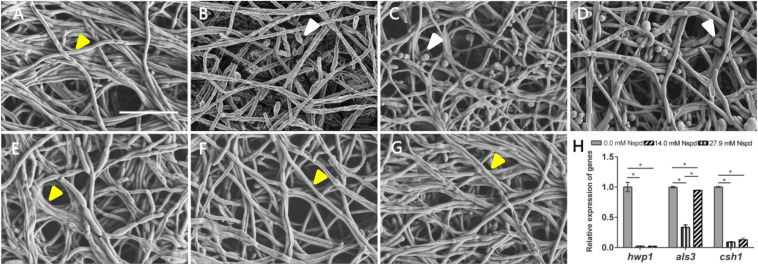
SEM images of *C. albicans* biofilms and virulence related gene expression influenced by Nspd on the *C. albicans* biofilms. **(A–E)** SEM images of *C. albicans* biofilms challenged by Nspd at various concentrations (0, 111.7, 55.9, 27.9, and 14.0 mM, respectively). **(F)** 80 μM fluconazole. **(G)** Vehicle (0.08% ethanol). More yeasts (indicated by white arrows) were formed in 111.7 mM **(B)**, 55.9 mM **(C)**, and 27.9 mM **(D)** Nspd groups. There were no yeasts but hyphae form of fungal cells (indicated by yellow arrows) in mature biofilms of blank control **(A)**, 14.0 mM Nspd group **(E)**, 80 μM fluconazole **(F)**, and vehicle **(G)** groups. Bar = 20 μm. **(H)** Virulence related gene expression of *hwp1*, *als3*, and *csh1* revealed by qRT-PCR on 48 h mature *C. albicans* biofilms. Data were presented in mean ± standard deviation; *p < 0.05.

The *C. albicans* biofilms formed on acrylic resin specimens were observed to assess biomass and fungal morphology by SEM (Figure 3). Different concentrations of Nspd could partially eliminate pre-formed *C. albicans* biofilm and this inhibitory effect on fungal biofilm gradually declined with the dilution of Nspd (Figures 3A,G). Also, there was more yeast form presented in Nspd groups when compared with blank control. While in 80 μM fluconazole, blank and vehicle controls, the proportion of yeast form seemed to have no difference.

Nspd seemed to be able to promote the expression of yeast phase. There existed higher proportion of yeast phase in pre-formed *C. albicans* biofilms in Nspd containing groups than that of blank control, especially in 111.7, 55.9, and 27.9 mM groups. Hypha and yeast are two different forms of *C. albicans*, and the morphological transition between hyphae and yeast forms is associated with the pathogenicity of *C. albicans.* Hypha, an important virulence attribute of *C. albicans*, shows more invasiveness compared to yeast and it promotes the host tissue penetration and leads to the establishment of systemic infections (Raut et al., 2013). It is reported that hyphal formation could be facilitated through a number of environmental factors such as serum or N-acetylglucosamine, neutral pH, CO_2_, body temperature, starvation and embedded growth conditions (Vesely et al., 2017). Nspd can promote hyphal development through regulating the pH of the medium (da Silva Dantas et al., 2016). Yet our result showed more yeast formation in high dosage of Nspd groups which seemed conflicts. Previous studied suggested that yeast formation was believed to represent the cell form that primarily involves in dissemination (Saville et al., 2003). We proposed that at high concentration of Nspd, fungi tended to switch to a smaller yeast form to escape from where the environment did not favor the survival of *C. albicans*.

### Nspd Reduced the Expression of Virulence-Related Genes

The expression of *hwp1* reduced to 2.2% and 2.3% in 27.9 mM Nspd and 14.0 mM Nspd groups when compared with blank control (*p* < 0.05) (Figure 3H). Also, the expression of *csh1* showed a similar situation to *hwp1* despite that *als3* had raised its expression at a concentration of 14.0 mM, which was slightly lower than that of the control group. Researchers had discovered the positive correlation between fungal hydrophobicity and biofilm formation in *C. albicans* (Silva-Dias et al., 2015). Also protein family is one of the most widely studied *C. albicans* virulence attributes and deletion of *als3* produces the greatest reduction in adhesive function. Moreover, als3 makes the largest contribution to adhesion to human cells (Lin et al., 2014). Those were consistent to our research findings. 27.9 and 14.0 mM Nspd had significantly decreased the expression of *csh1* (Figure 3H) resulting in obvious biomass volume reductions (Figure 1A). Our research also made an addition to the research of correlation between fungal hydrophobicity and biofilm metabolic activity in *C. albicans*, where Silva-Dias and co-workers did not find any correlation. Due to the difference in culture condition/substratum and fungal form, we were able to display a dramatic metabolic reduction in 27.9 and 14.0 mM Nspd groups where hydrophobicity had been down-regulated significantly (Figure 2H).

SEM images of mature *C. albicans* biofilm challenged by for 48h showed that there were less biofilms biomass and less proportion of hyphal formation. Silva-Dias claimed that yeast form of *C. albicans* had weaker adhesion than its hyphal form. This might explain our SEM and biomass findings. Challenged by Nspd, there were more yeasts (Figure 3B) than hyphae (Figures 3A,G) in *C. albicans* biofilm, confirmed by SEM observations; the biomass was less reduced at low Nspd dosage and at high dosage (Figure 1A).

Taken together, we claimed that Nspd was effective in treating a mature fungal biofilm via its downregulating on virulence related genes. In regenerative medicine and clinical practice, fungal related infection control often co-exists with the need for tissue regeneration. Stem cell therapy being one of promising cell source has become a research hot topic. The regenerative potential and clinical application of stem cells have been extensively studied. Accumulating evidence has proved that vascularization is an important step toward the success of tissue regeneration (Wang et al., 2010). Petri and co-works claimed that stem cell concentrates can be an alternative to segmental bone regeneration for long-bone defects are larger than 3 cm (Petri et al., 2013). Critical size long bone defect could be completely re-grown with the aid of vascularization. DPSCs were able to differentiate into functional endothelial cells. In this work, we aimed to test whether the presence of Nspd would affect the tissue regeneration potency of DPSCs.

### DPSCs Identification and Lineage Differentiation

**FIGURE 4:**
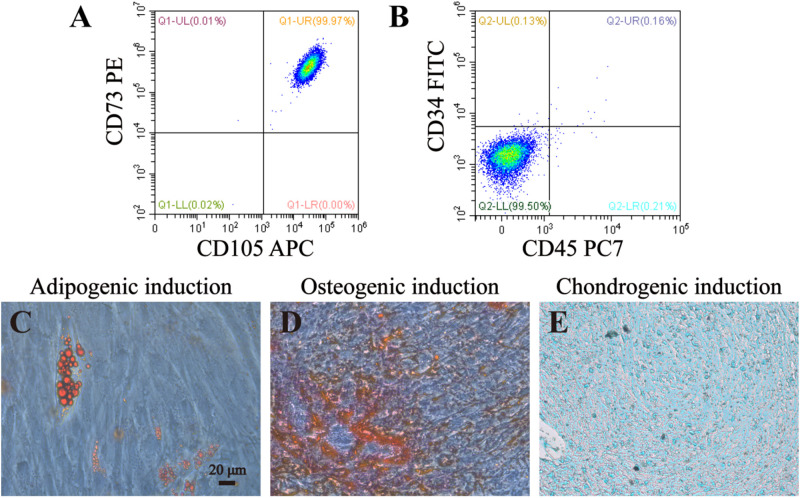
Identification and lineage differentiation of DPSCs. **(A,B)** Flow cytometry confirmed that DPSCs isolated and cultured in present study positively expressed surface markers of mesenchymal stem cells (CD73 and CD105), and negatively expressed surface markers of hematopoietic stem cells (CD34 and CD45). **(C–E)** Multiple differentiation potency of DPSCs used in this study was assessed by osteogenic, adipogenic and chondrogenic experiments. Fat droplets (red in **C**), calcified nodules (red in **D**), and chondrocytes (blue in **E**) were presented after 21 or 28 days inductions. Bar = 20 μm.

In order to identify DPSCs, flow cytometry and multilineage differentiation were performed. According to our result, DPSCs could express MSC-like marker CD73 and CD105, but negatively expressed CD34 and CD45, which is two of the surface markers of hematopoietic stem cells (Figures 4A,B). These data confirmed that DPSCs did have MSC-like immunophenotype. The results of multilineage differentiation confirmed a good regenerative potency upon adipogenic, osteogenic and chondrogenic inductions (Figures 4C–E).

### DPSCs Could Proliferate and Differentiate Normally With the Presence of 0.63 mM Nspd

**FIGURE 5:**
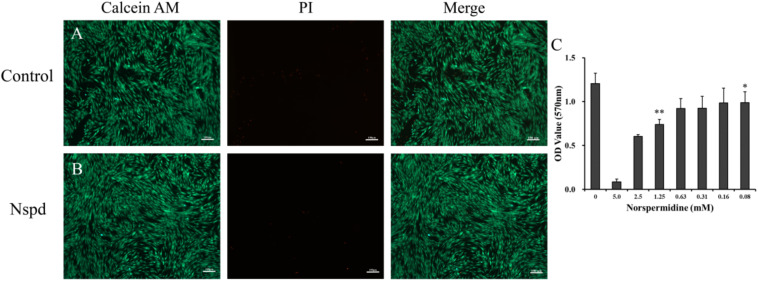
Live/dead staining of DPSCs with and without Nspd and the cytotoxic of Nspd determined by MTT assay. **(A)** DPSCs cultured with α-MEM medium, the control. DPSCs were in spindle shapes and were well aligned in typical pattern. **(B)** DPSCs cultured with α-MEM medium containing 0.63 mM Nspd. Similar to those in the control, DPSCs were densely aligned. Calcein AM (green) was used to stain live stem cells and PI (red) was used to stain dead stem cells. Bar = 100 μm. **(C)** To identify an optimal concentration of Nspd for DPSCs study, Nspd at various concentrations (0, 5.0, 2.5, 1.25, 0.63, 0.31, 0.16, and 0.08 mM, respectively) were used to culture DPSCs for 24 h. Live/dead cell viability for DPSCs. MTT assay was used to assess the viability and proliferation rate of DPSCs with presence of Nspd. Data were presented in mean ± standard deviation. *represented 0.08, 0.16, 0.32, and 0.64 mM of Nspd were significantly different from control group, (0 mM). **represented the result was significantly different from the 0.63 mM Nspd group.

Fluorescent microscopic study on the DPSCs with the presence of Nspd showed that the morphology and vitality of the stem cells were not influenced compared with blank control (Figures 5A,B). Metabolic activity of DPSCs cultured with and without Nspd was revealed by MTT assay. In this study, we discovered that the metabolism of DPSCs were tolerant to Nspd at 0.63 mM and lower levels. When DPSCs were cultured with the highest tolerable 0.63 mM Nspd level, the vitality of DPSCs was reduced by 23.5% compared with that of the control. Although there was statistical difference between 0.63 mM group and control group (*p* < 0.05) (Figure 5C), the stem cells subject to 0.63 mM Nspd still held a fine vitality and proliferation ability. Cell viability varied with cell types. Cardile et al. reported that 1 mM Nspd reduced the viability of human keratinocytes to 70% and human fibroblasts to 80% compared with blank control in 24 h *in vitro* culture (Cardile et al., 2017). In our study, 1.25 mM Nspd reduced the viability of DPSCs to 60% compared with blank control, where it was significantly lower than that of 0.63 mM Nspd. 0.025 and 0.1 mM Nspd could inhibit some breast cancer cell lines in 24 h culture (Silva T. M. et al., 2013). Taken together, tissue cell tolerance of Nspd decreased in following order: fibroblasts > keratinocytes > DPSCs > some breast cancer cells.

**FIGURE 6:**
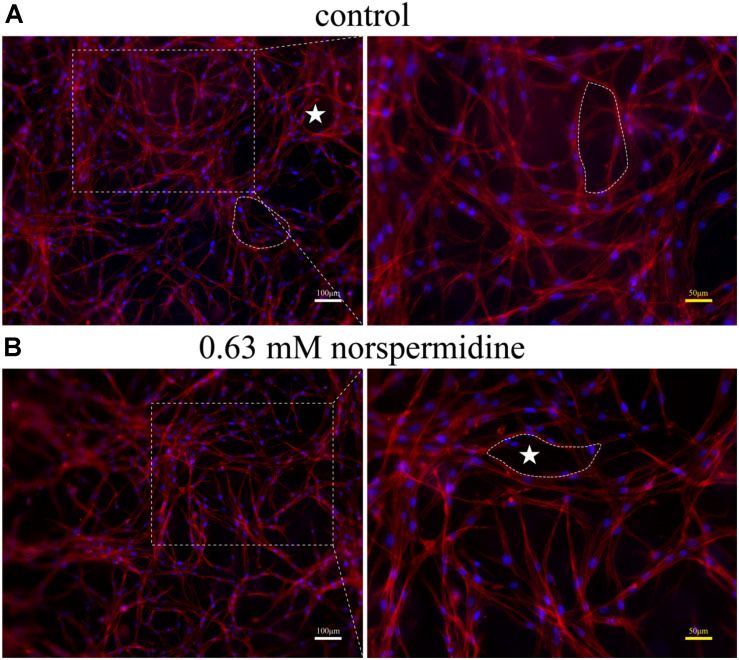
Fluorescence images of capillary-like structure of DPSCs after 7 day angiogenic induction with and without Nspd cultured in GelMA. Differentiated DPSCs were stained with Phalloidin-TRITC (red) to specifically display F-actin, a capillary-like network structure and DAPI (blue) to display cell nuclei. **(A)** Inducted by growth medium as control; **(B)** Induced by media with 0.63 mM Nspd. In both groups, capillary-like network was observed (white stars). White bar = 100 μm, and yellow bar = 50 μm.

Further, when DPSCs were undergone angiogenic differentiation, 0. 63 mM Nspd did not show obvious influence on differentiated cells and capillary-like network formation was observed (Figure 6). In both control and 0.63 mM Nspd groups, we could see well differentiate cells stretching and forming capillary-like structures in the co-cultured with GelMA hydrogels. Together, we defined a tolerable range of Npsd (0.63–0.08 mM) for *in vitro* cell culture of DPSCs; proved that DPSCs could be induced into endothelia like cells with the presence of Nspd at safe dosage.

**FIGURE 7:**
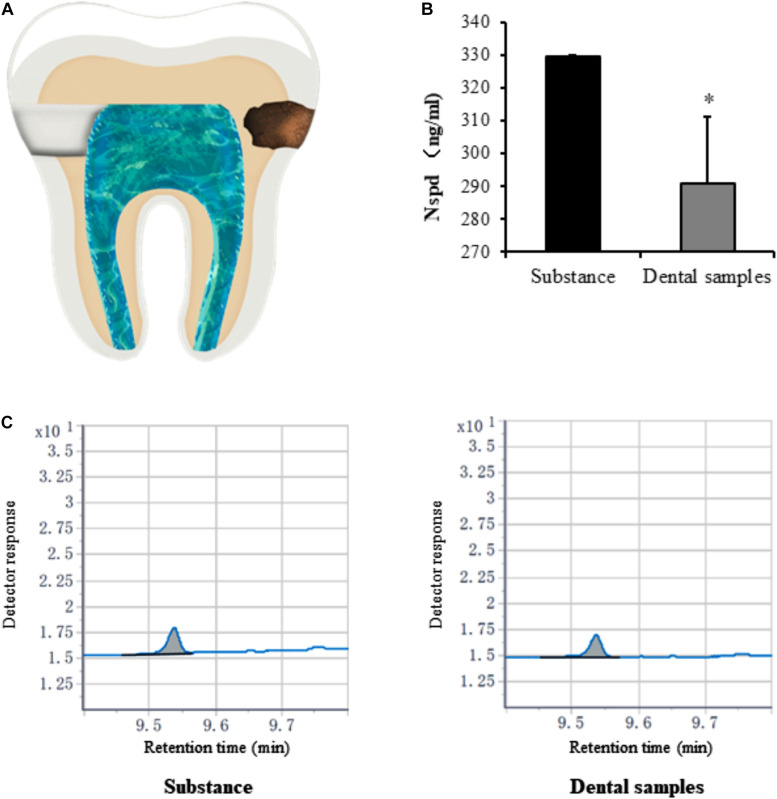
Quantification of norspermidine in dental caries model *in vitro*. **(A)** The opening location of pulp chamber (left side) and Cavity of tooth (right side) in dental caries model. **(B)** Nspd was quantified according to the peak area detected with Agilent GC system. * represented dental samples group was significantly different from substance group. Data were presented in the mean ± standard deviation; *p < 0.05. **(C)** Detector response of Nspd. Substance group was diluted 250 times with 0.63 mM Nspd, and the dental sample was also diluted 250 times.

Caries model experiment indicated that only small amounts of Nspd enter (significantly less than 0.63 mM) the pulp cavity (Figures 7B,C). Considering the clinical treatment will be taken to seal the dentinal tubules and other means, we assessed that Nspd application won’t cause any side effect to pulp tissue regeneration.

## Conclusion

Our project was the first to study the effect of Nspd on a 48 h old mature *C. albicans* biofilm with additional contribution to the mechanism study on the virulence characteristics of *C. albicans*. Our results showed that 111.7–14.0 mM Nspd showed a dose- inhibitory effect on this mature fungal biofilm. High concentration of Nspd (111.7 and 55.9 mM) inhibit the fungal viability in the mature biofilms and reduced the biomass and metabolic activity of these biofilms significantly. Medium concentration of Nspd (27.9 and 14.0 mM) displayed a moderate fungicidal effect and significantly suppressed the expression of virulence related genes. 111.7–27.9 mM Nspd restrained the production of hyphae form in mature biofilms. In biosafety study of Nspd with DPSCs, Nspd at 0.63 mM and lower was safe for DPSCs based angiogenic application. Based on our results, Nspd seemed to be a potential new drug against infections caused by *C. albicans* biofilms especially when dental pulp vitality or dental pulp regeneration was considered.

## Data Availability Statement

The raw data supporting the conclusions of this article will be made available by the authors, without undue reservation.

## Author Contributions

KZ, YH, and QY conceived the idea. The work was done by YH, YC, KZ, LL, YX, and FT. YZ, FH, AA, and ZX analyzed the data. KZ, QH, and QY discussed and interpreted the results. YH, YC, YS, and KZ wrote the manuscript. QH and QY critically revised the manuscript. All authors contributed to the article and approved the submitted version.

## Conflict of Interest

The authors declare that the research was conducted in the absence of any commercial or financial relationships that could be construed as a potential conflict of interest.
